# Trained Immunity and Trained Tolerance: The Case of *Helicobacter pylori* Infection

**DOI:** 10.3390/ijms25115856

**Published:** 2024-05-28

**Authors:** Maria Pina Dore, Giovanni Mario Pes

**Affiliations:** 1 Dipartimento di Medicina, Chirurgia e Farmacia, University of Sassari, Clinica Medica, Viale San Pietro 8, 07100 Sassari, Italy; gmpes@uniss.it; 2 Department of Medicine, Baylor College of Medicine, One Baylor Plaza Blvd, Houston, TX 77030, USA

**Keywords:** trained immunity, *Helicobacter pylori*, gastritis, immunocompetent cells

## Abstract

Trained immunity is a concept in immunology in which innate immune cells, such as monocytes and macrophages, exhibit enhanced responsiveness and memory-like characteristics following initial contact with a pathogenic stimulus that may promote a more effective immune defense following subsequent contact with the same pathogen. *Helicobacter pylori*, a bacterium that colonizes the stomach lining, is etiologically associated with various gastrointestinal diseases, including gastritis, peptic ulcer, gastric adenocarcinoma, MALT lymphoma, and extra gastric disorders. It has been demonstrated that repeated exposure to *H. pylori* can induce trained immunity in the innate immune cells of the gastric mucosa, which become more responsive and better able to respond to subsequent *H. pylori* infections. However, interactions between *H. pylori* and trained immunity are intricate and produce both beneficial and detrimental effects. *H. pylori* infection is characterized histologically as the presence of both an acute and chronic inflammatory response called acute-on-chronic inflammation, or gastritis. The clinical outcomes of ongoing inflammation include intestinal metaplasia, gastric atrophy, and dysplasia. These same mechanisms may also reduce immunotolerance and trigger autoimmune pathologies in the host. This review focuses on the relationship between trained immunity and *H. pylori* and underscores the dynamic interplay between the immune system and the pathogen in the context of gastric colonization and inflammation.

## 1. Introduction

Trained immunity (TI) is a concept in immunology in which innate immune cells, such as monocytes and macrophages, exhibit enhanced responsiveness and memory-like characteristics following initial contact with a pathogenic stimulus. The increased response can result in effective immune defense upon subsequent contact with the same pathogen. *Helicobacter pylori* (*H. pylori*), a Gram-negative micro-aerophilic flagellated bacterium, specifically colonizes the gastric mucosa. The stomach provides a stressful environment for an effective immune response, as both the pH and the oxygen tension are typically low. Functionally, the infection is primarily of the surface mucosa and is thus technically outside of the body. *H. pylori* is largely able to evade defense mechanisms and establish persistent infections. The intensity of the response depends in part on the ability of the pathogen to manipulate the response mechanisms of the host’s immunocompetent effector cells, including macrophages and natural killer (NK) cells. Increasing evidence obtained via in vitro and in vivo experiments in which phagocytic cells are exposed to *H. pylori* suggests that *H. pylori* can rewire intracellular signaling pathways to enhance the development of persistent infections.

*H. pylori* infections have undergone co-evolution with humans [1], allowing the pathogen ample opportunity to develop highly efficient mechanisms to evade clearance by the host’s immune system. This is reflected in the fact that (i) the bacterium can trigger both an acute form of gastritis, similar to that of other pathogens, as well as a chronic persistent form, which increases the risk of peptic ulcers and gastric cancer; (ii) *H. pylori* may also induce immune tolerance in the host, enhancing the persistence of the infection; and (iii) rarely, the onset of autoimmunity [2]. A better understanding of innate immune dampening during *H. pylori* infection should favor the development of an improved approach resulting in more efficient prophylactic and therapeutic measures.

The ability of *H. pylori* to manipulate the host immune system to evade recognition and clearance and achieve a long-term colonization of the stomach is a multifactorial adaptive response that may involve several strategies (Table 1).

While there is evidence that *H. pylori* may downregulate both host innate and acquired immune mechanisms, the former phenomenon is relatively less investigated. The present review aims to provide an updated overview of the mechanisms involved.

## 2. General Intracellular Mechanisms Involved in Trained Immunity

Although immune memory is usually ascribed to adaptive immune cells, there is evidence that cells typically involved in innate immunity, such as macrophages and NK cells, also retain traces of early contact with pathogens and modify their reactivity accordingly.

The most common cell types involved in TI are innate lymphoid cells (ILCs), which include cytotoxic (NK cells) and non-cytotoxic ILCs (ILC1, ILC2, and ILC3). One feature of these cells is that they express cytokine receptors, undergo transcriptional modifications, and ignite the inflammatory cascade. However, unlike T and B cells, ILCs do not produce the antigen receptors necessary to confer antigen specificity [10]. Instead, the binding of these cells with a training agent results in metabolic changes, which are regulated by epigenetic modifications, making them capable of reacting more efficiently upon subsequent contact with the same or similar pathogen. Various training agents exist and have been systematically included in the TI database (TIDB) [11]. Although TI is usually associated with the induction of memory, thus enabling a stronger immune response in the case of subsequent infection, it is also likely that repeated contact with the same pathogen can result in a modification of the functional state of innate cells in a tolerogenic direction.

**Figure 1:**
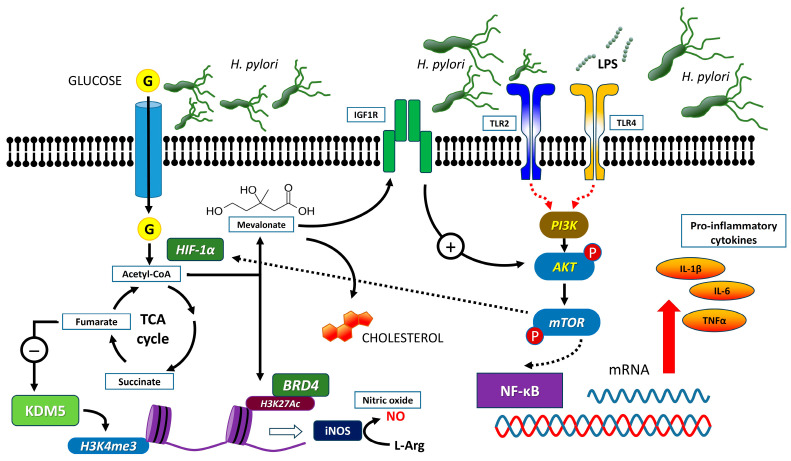
Schematic representation of the mechanisms involved in the trained immunity induced by *Helicobacter pylori* (for the explanation see the text).

Training mechanisms are intricate and were studied for the first time by Netea et al. using β-glucan, one of the constituents of the *Candida albicans* cell wall [12]. This molecule binds to the TLR2 and TLR4 receptors of human monocytes, and subsequent contact with agents capable of activating these receptors can activate immunocompetent cells. This results in a reprogramming consisting of metabolic and epigenetic changes, including increased histone methylation and acetylation in promoters and enhancers of pro-inflammatory genes [9]. Training agents, such as β-glucan and others, induce stable metabolic modifications such as upregulation of the tricarboxylic acid (TCA) cycle via the Akt/mTOR signaling pathway, increasing the production of acetyl-CoA, succinate [13], fumarate [14], and mevalonate (a precursor of cholesterol synthesis) [15,16]. Acetyl-CoA promotes the acetylation of histones and the remodeling of chromatin architecture. These events are associated with enhanced expression of several key epigenetic markers, such as histone-3 lysine-27 acetylation (H3K27Ac) and histone-3 lysine-4 methylation (H3K4me1) (Figure 1). These changes enhance or repress the function of proinflammatory genes, including tumor necrosis factor (TNF)-α and IL-6 [9,17], which are involved in the activation of the innate immune system. Moreover, acetyl-CoA indirectly stimulates cholesterol biosynthesis, and one of the intermediate products, mevalonate, potently amplifies training by interacting with the insulin-like growth factor 1 receptor (IGF1R) to further stimulate the Akt pathway. Fumarate inhibits the histone demethylase KDM5 and thereby promotes trimethylation of histone H3 on the K4 residue (H3K4me3) [18], ultimately increasing cytokine release during induction of TI.

## 3. Interactions of *H. pylori* with Cells of the Host

The gastric epithelial cells are the first line of defense against *H. pylori* infection [19]. They activate immunocompetent cells through TLRs, which act as sensors for the bacterium. Through TLR pathways, *H. pylori* stimulates immune activation with the consequent release of proinflammatory cytokines, including IL-1β, IL-6, and TNF-α [20]. However, after a transient acute phase, *H. pylori* infection may become persistent owing to the capacity of the bacterium to alter the response mechanisms of immunocompetent effector cells, including macrophages [21,22,23], NK cells, which are phenotypically identified by the expression of the adhesion molecule CD56 [24], and T lymphocytes [25]. In addition, Zhang et al. demonstrated in animal models that prolonged exposure to the bacterium desensitizes mucosal epithelial cells to TLR2/6 heterodimers, resulting in the downregulation of the PI3K/NF-κB and MAPK pathways [26]. This leads to an inefficient host immune response, which facilitates *H. pylori* immune escape. Instead, the use of TLR2/6 heterodimer agonists in vitro restores the sensitivity of epithelial cells to *H. pylori* [26]. It follows that the use of these molecules could prove to be valid immunotherapeutic agents capable of limiting the tolerogenic mechanisms underlying the persistence of *H. pylori* infection in the stomach.

During infection by *H. pylori*, an increase in circulating monocytes, including those present in the gastric mucosa, is observed as a result of a chemotactic stimulus [27,28]. The accumulation of these innate immune cells, which release proinflammatory cytokines and chemokines, reflects an early inflammatory response aimed at clearing the bacterium. Upon *H. pylori* infection, macrophages are polarized into an M1 phenotype characterized by the ability to release cytokines to kill bacteria [29]. They also produce nitric oxide (NO) through the reaction catalyzed by the inducible nitric oxide synthase (iNOS), an event mediated by the epigenetic transcriptional regulator bromodomain-containing protein 4 (BRD4) [30,31] (Figure 1). Initially, *H. pylori* reshapes immunocompetent cells in a pro-inflammatory and oxidative direction with the cooperation of macrophage BRD4 and the glycolysis regulator HIF-1α. However, as the infection progresses, a decreased number and activity of monocytes and NK cells may ensue [32]. This may be due to the fact that *H. pylori*, despite favoring the activation of immunocompetent cells, also powerfully stimulates apoptosis, which reduces the inflammatory response and causes the transition from an acute reaction to persistent chronic infection [32]. In addition, *H. pylori* can inhibit its own phagocytosis by monocytes present at a site of infection through a mechanism involving the intervention of components of the type IV secretion system [33]. Using immunofluorescent staining and advanced microscopy techniques, it was demonstrated that the antiphagocytic action depends on the presence of a *cag* pathogenicity island (*cag*PAI). Approximately 80% of bacteria engulfed in macrophages are found to have this variant, and PAI-deleted mutants are rapidly ingested by phagocytic cells [22]. Attempts to make use of TI to stimulate cells of the immune system to prevent the persistence of *H. pylori* in the gastric mucosa have not been very numerous and have mainly used β-glucan, lipopolysaccharide (LPS), and the bacillus Calmette-Guérin (BCG—a live attenuated strain of *Mycobacterium bovis* developed by Calmette and Guérin to prevent tuberculosis and other mycobacterial infection), and have had limited success.

### 3.1. β-Glucan

The first studies aimed at clarifying the mechanisms involved in TI used β-glucan, a polysaccharide isolated from the cell wall of *C. albicans* [34]. The immunomodulatory properties of β-glucan depend both on its physical properties, such as size, conformation, and solubility, and on the cells with which it interacts [35]. Like many bacterial constituents, this molecule acts as a pathogen-associated molecular pattern (PAMP) and, as such, is recognized by pattern-recognition receptors (PRRs) expressed on innate immune cells. Through binding to the dectin-1 receptor present on monocytes, macrophages, and dendritic cells, β-glucan activates the intracellular Akt/mTOR/HIF1α pathway, which ultimately induces metabolic changes, including upregulation of glycolysis and TCA, the final mediators of TI [17] (Figure 1). Based on in vitro studies suggesting that β-glucan acts as an immune stimulator capable of activating macrophages to clear *H. pylori*, a double-blind, randomized clinical trial examined its efficacy as an oral treatment of long-lasting gastritis and obtained beneficial effects [36]. This study also highlighted decreased blood glutathione (GSH) levels, which, however, could be the consequence of a reduced dietary availability of amino acid precursors of glutathione due to the inflammation of the gastrointestinal tract [36].

### 3.2. Lipopolysaccharide

**Figure 2:**
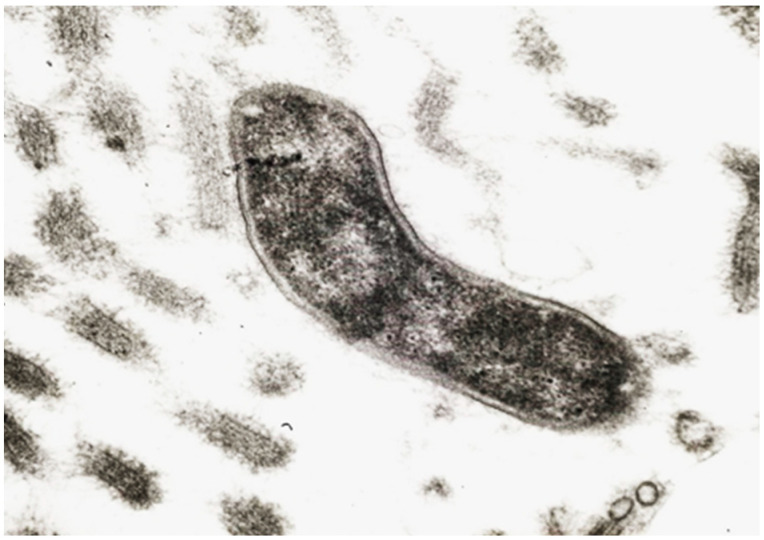
Electron micrograph showing *H. pylori* with its LPS layer clearly visible, adhering to the gastric mucosa.

The LPS, a membrane component of Gram-negative bacteria, can elicit TI through several signaling pathways by upregulating genes that counteract secondary infection [37]. *H. pylori* LPS binds to the TFF1 protein of gastric mucus [38] but, unlike the LPS of most Gram-negative bacteria, is poorly immunoreactive [39,40] (Figure 2). There is evidence that LPS stimulation can reprogram innate immune cells through the MAPK/p38 signaling pathway [41] as well as via epigenetic modifications.

Lipopolysaccharide induces transcriptional changes that result in decreased release of proinflammatory cytokines [42], such as interleukin (IL)-6, TNF-α, and IL-1β, while the release of the anti-inflammatory cytokine IL-10 is increased [43]. When primary human monocytes are infected with *H. pylori*, the TI mechanisms elicit hyperresponsiveness to subsequent LPS challenges [44]. It has also been demonstrated that monocyte priming by *H. pylori* induces innate immune memory characterized by intense nuclear translocation of NF-κB proteins, which elicits a vigorous response to the persistence of infection [45]. LPS priming induces methylation at H3K4 in macrophages [46], as well as increased phosphorylation of transcription factors, such as ATF7 [47], resulting in a stable decrease of repressive H3K9me2. Moreover, as in the case of β-glucan, the LPS challenge has been shown to upregulate TCA, thus increasing succinate levels [48].

However, as the infection persists, immunotolerance prevails, as revealed by the presence of histone markers (H3K27ac and H3K4me1) at promoters or enhancers of genes responsible for phagocytosis [31]. Coletta et al. [49] recently reported that the *H. pylori* LPS heptose metabolite ADP-heptose, which exhibits an important role in monocyte and macrophage activation [50], is a major factor in reducing the expression of antigen-presenting surface molecules of macrophages. In animal models, the addition of heptose-ADP, as well as live bacteria or heptose-enriched *H. pylori* lysates, has been shown to result in an upregulation of a non-coding miRNA (miR146b) in macrophages [51]. This, in turn, downregulates the expression of CIITA (transactivator of HLA-II protein expression), whereas *H. pylori*, without the added heptose, is unable to complete downregulation. Therefore, it has been suggested that, through the production of the heptose metabolites, the LPS of *H. pylori* is able to block communication between the innate and adaptive systems despite the ongoing activation of the macrophage system [52]. These findings provide a better insight into the mechanisms by which proinflammatory and immune processes of phagocytic cells evade following exposure to bacteria.

### 3.3. The Role of Cholesterol

Cholesterol is a polycyclic unsaturated alcohol deeply involved in the structure, function, and membrane fluidity of eukaryotic cells. It is an integral constituent of membrane lipid rafts involved in the downstream signaling of cells [53]. Steryl glucosides, which are frequently found in plants and fungi, are rare in bacteria [54]. Thus, *H. pylori*, like most prokaryotes, cannot synthesize cholesterol and must obtain it from the host’s cells. There is evidence that *H. pylori* is able to modify the host’s cholesterol to adhere to epithelial cells and inhibit its own phagocytosis [55,56].

#### 3.3.1. Enzymatic Glycosylation of Cholesterol

The auxotrophy of *H. pylori* for cholesterol requires the bacterium to move close to sterol-rich cells during infection. *H. pylori* can sense concentrations of cholesterol that are 20 times lower than those present in human serum [55]. This tropism to cholesterol is not seen in non-motile mutants. Confocal microscopic analysis in animal models demonstrated that the bacterium colocalizes with the GM1 ganglioside, a typical component of cholesterol-rich lipid rafts [57]. A few hours after infection, cells in contact with the bacterium show increased levels of glycosylated cholesterol derivatives [55]. Enzymatic glycosylation of cholesterol by *H. pylori* is catalyzed by cholesterol-α-glucosyltransferase, which was demonstrated to be essential for bacterial adherence to cells [56]. *H. pylori* adhesion to cells is promoted by a number of molecules found in the membrane lipid rafts, including PAR1/MARK, Lewis antigens, and integrin α5β1 [58]. More importantly, glycosyl-cholesterol inhibits phagocytosis and phagosome maturation in mouse macrophages [55], and it is assumed that this interaction may also occur in humans. The interaction between APC and T cells is strongly increased when *H. pylori* is incubated with cholesterol before infecting the animal, whereas this response is inhibited by antibodies blocking class I or II molecules of the HLA system [55]. Transcriptome analysis of the gastric mucosa of animals previously treated with cholesterol and later infected by *H. pylori* demonstrates upregulation of interferon-γ (IFNγ)-regulated genes compared to animals with untreated mucosa [59]. However, not all glycosidic derivatives synthesized by the bacterium have the same effect, since loading *H. pylori* with cholesteryl-β-glucoside promotes normal phagocytosis by macrophages, whereas this does not occur in the case of *H. pylori* loaded with cholesteryl-α-glucoside. Therefore, the latter, unlike the former, has a protective (immune evasion) action for the bacterium. The association of *H. pylori* with lipid rafts is particularly intriguing: These are dynamic cholesterol-rich membrane subdomains, containing specific proteins that play crucial roles in signal transduction, membrane trafficking, and protein sorting [60]. Among the latter, there are pattern recognition receptors (PRRs), such as toll-like receptors (TLRs) and C-type lectin receptors (CLRs), which are critical for detecting pathogen-associated molecular patterns (PAMPs). The presence of these receptors in lipid rafts facilitates their interaction with microbial components and the initiation of the downstream signaling cascade. Lipid rafts are involved in the recruitment and activation of various immune cells, including macrophages and dendritic cells. Upon activation, these cells undergo dynamic changes in membrane lipid composition and organization, leading to the redistribution of lipid rafts and the clustering of immune receptors at the site of pathogen entry. The ability of *H. pylori* to disrupt lipid rafts in macrophages and NK cells may be an important mechanism to escape immune detection and promote survival and replication [60,61]. Given that the spatial organization of lipid rafts in macrophages is known to be under epigenetic control [62], it would be interesting to investigate whether *H. pylori* is able to train immune cells to tolerate the pathogen following sustained interactions with the host.

#### 3.3.2. Mevalonate

Mevalonate, which is synthesized from acetyl CoA as a training agent, increases cytokine release upon restimulation [63]. The TI induced by mevalonate drives metabolic and epigenetic modifications, enabling circulating monocytes to maintain a sustained response against the pathogen through the release of IL-1β and IL-32 [63]. Epigenetic reprogramming by mevalonate acts in a similar way as β-glucan, and mevalonate inhibitors are promising agents that might be used in the future against *H. pylori* infection [64]. On the other hand, mevalonate-dependent TI may cause the innate immune system to mount a weaker immune response upon persistent *H. pylori* infection [65].

### 3.4. Bacillus Calmette-Guérin

BCG was found to be one of the most important agents capable of driving TI [66]. BCG acts by binding to both TLR2 and TLR4, thereby modulating intracellular signaling pathways [67]. Several in vitro studies ascertained that monocytes treated with BCG may activate the mammalian target of the rapamycin (Akt/mTOR) pathway, leading to the upregulation of metabolic pathways such as glycolysis and oxidative phosphorylation [14]. An additional mechanism involved in TI of monocytes exposed to BCG is the nucleotide-binding oligomerization domain 2 (NOD2) pathway activated via TLR2/4, which can induce the cytokines IL-1β, IL-6, and TNF-α. In fact, NOD2 blockade in BCG-stimulated monocytes significantly reduces TNF-α release upon reinfection. IL-1β appears to be an especially key cytokine induced by BCG training: In BCG-stimulated monocytes, IL-1β levels increased after restimulation, conferring non-specific protection against the infection. The release of cytokines by BCG-treated monocytes subsequently challenged with *H. pylori* is regulated by epigenetic mechanisms. Notably, BCG was found to increase the H3K27Ac marker in several signaling pathways involved in the immune response, such as PI3K/AKT [68]. In general, epigenetic changes in signaling pathways induced by BCG training are responsible for enhanced innate immune responses upon restimulation. Given that these mechanisms are not exclusive to monocytes but are also detected in other immune cell types, it is possible that an evolutionarily conserved core component exists.

Since BCG vaccination has been demonstrated to possess therapeutic effects against Gram-negative infections, it has been hypothesized that BCG may offer immune cross-protection against *H. pylori* infection as well [69]. A clinical trial investigated the relationship between BCG vaccination and protection against *H. pylori* [70], and the results showed no occurrence of *H. pylori* infection in the arm of patients vaccinated with BCG compared with the arm treated with placebo, although the small sample size made this difference not statistically significant. A study in an animal model demonstrated that BCG treatment was able to reduce the adhesion of *H. pylori* to gastric cells, although the effect was mostly attributed to the ability of BCG to modulate mucin production, thus inhibiting gastric colonization by *H. pylori* [69]. Thus, further research is necessary to evaluate the long-term efficacy of BCG treatment in order to optimize the cellular response for therapeutic purposes.

BCG can also function to inhibit cell signaling or alter epigenetic modifications required to induce TI. For instance, the anti-inflammatory cytokines IL-37 and IL-38, as well as hydroxychloroquine, have demonstrated inhibitory effects against TI [70]. Taken together, TI in *H. pylori* infection, in addition to increased inflammation, may also imply immunotolerance, namely reprogramming of innate immune cells and facilitating the persistence of infection.

## 4. Trained Immunity and *H. pylori* Comorbidities

The existence of TI mechanisms in *H. pylori* infection means that the long-term establishment of immunological tolerance can have consequences that go beyond gastric involvement and can involve other organs and systems. It is known that *H. pylori* infection is sometimes associated with extragastric manifestations that may have an autoimmune component [71]. Various *H. pylori* antigens have a high homology with those of molecules of human tissues, including the β subunit of urease, which is structurally similar to the β subunit of ATPase in gastric parietal cells. This could explain the occurrence of cross-reactivity mediated by molecular mimicry [72]. Among the toxins released by *H. pylori*, the vacuolating cytotoxin gene A (*VacA*) exerts multiple functions including the regulation of the host immune system [73]. Although *VacA* is able to induce an NF-κB-mediated inflammatory response, resulting in upregulation of the chemokine IL-8, this toxin is also able to alter immune tolerance by inhibiting dendritic cell activation, thereby facilitating the persistence of the infection but increasing the susceptibility to autoimmune processes. Preclinical evidence has been provided that administering *H. pylori* whole cell extract to allergen-sensitized mice conferred protection against asthma, which was not observed by treating with other Gram-negative enteric pathogens such as *E. coli* or *Salmonella typhimurium* [74]. It has previously been shown in animal models that infection with *H. pylori*, isolated from humans, confers protection against allergen-induced asthma in C57/BL6 mice, especially in younger animals [75]. According to the authors, these results suggested a specific tolerogenic reprogramming of dendritic cells exposed to *H. pylori* in vitro and in vivo and the induction of highly suppressive regulatory T cells [75,76]. Subsequent experiments, conducted by the same authors, again in animal models, demonstrated how extracts of *H. pylori* (γ-glutamyl transpeptidase, GGT, and vacuolating cytotoxin, *VacA*, positive), administered orally or intraperitoneally, induced tolerability by preventing bronchopulmonary hyperreactivity [74]. Given these observations, the authors concluded that the administration of pure GGT and *VacA* from *H. pylori* is sufficient for the protection and prevention of allergic asthma.

## 5. Discussion

*H. pylori* stands out among other Gram-negative bacteria for its ability to modify the host’s defensive response in a direction favorable to its survival. During the early stages of infection by *H. pylori*, monocytes increase the expression of both pro- and anti-inflammatory cytokines. Subsequently, these cells release only anti-inflammatory cytokines, which ensure the survival of the bacterium inside the stomach [44]. As a result, *H. pylori* is able to colonize the stomach permanently and can exert its pathogenic effect for many years. It is currently believed that this peculiarity of the bacterium is due to a clever reprogramming of the host’s signal systems in the context of TI, which, in this case, must rather be called a form of trained tolerance. We have seen how, as a secondary effect of this weakening of the host’s immune system, the general mechanisms of immunosurveillance in the host can be compromised, which consequently can trigger autoimmunity phenomena [71]. At the same time, knowledge of the role of TI in the case of *H. pylori* infection could be useful in the prevention or treatment of this infection by boosting the immune protective effects and disadvantaging the tolerogenic ones in several cell types.

## 6. Conclusions

In conclusion, this review summarizes how *H. pylori* infection is able to trigger a TI through a number of bacterial and host factors in a mutual interaction, favoring the chronicity and persistence of the infection. On the other hand, the immunomodulatory capabilities of the bacterium could be used for target therapies with *H. pylori*-specific tolerization, for example, in allergies, as proved in animal models by some authors. In the meantime, preventing gastric colonization by *H. pylori*, which is a class I carcinogen, and treating the infection is mandatory. However, until we can transfer this type of therapy to humans, preventing gastric colonization by *H. pylori* and treating the infection are mandatory.

## Figures and Tables

**Table 1:** Strategies adopted by *H. pylori* to evade the immune system.

	Mechanism	Reference
(i)	Adaptation of *H. pylori* to the acidic gastric environment by producing urease, an enzyme that hydrolyzes urea to ammonia to neutralize gastric pH, and enabling the bacterium to survive and multiply.	[3]
(ii)	*H. pylori* expresses adhesins and other surface molecules enabling it to adhere tightly to the gastric epithelial cells. This forms a biofilm-like layer shielding the bacterium from immune surveillance and clearance.	[4]
(iii)	Antigenic variation of the bacterium by altering the expression of surface antigens, such as the blood group antigen-binding adhesin (BabA) and the sialic acid-binding adhesin (SabA), which bind to gangliosides expressed in epithelial cells of the human stomach.	[5]
(iv)	*H. pylori* can form biofilms, i.e., structured communities of bacteria encased in a self-produced matrix. Biofilms provide protection against antimicrobial agents and immune cells, allowing *H. pylori* to persist and survive in the gastric mucosa.	[6]
(v)	The bacteria can invade gastric epithelial cells and reside intracellularly, where they may be protected from immune surveillance and antibiotic treatment.	[7]
(vi)	*H. pylori* can reshape the host immune response to its advantage by activating regulatory T cells (Tregs) and suppressing pro-inflammatory responses, leading to a state of immune tolerance or immunosuppression in the gastric mucosa.	[8]
(vii)	*H. pylori* can alter the expression of various host’s immune genes and cytokines, thus dampening the immune response.	[9]
